# Inflammatory cytokine expression in Fabry disease: impact of disease phenotype and alterations under enzyme replacement therapy

**DOI:** 10.3389/fimmu.2024.1367252

**Published:** 2024-08-21

**Authors:** Yujing Yuan, Yawen Zhao, Fan Li, Chen Ling, Yuan Wu, Wei Ma, Zhaoxia Wang, Yun Yuan, Hongjun Hao, Wei Zhang

**Affiliations:** ^1^ Department of Neurology, Peking University First Hospital, Beijing, China; ^2^ Department of Ophthalmology, Peking University First Hospital, Beijing, China; ^3^ Department of Cardiology, Peking University First Hospital, Beijing, China; ^4^ Beijing Key Laboratory of Neurovascular Diseases, Beijing, China; ^5^ Department of Neuroimmunity, Peking University First Hospital, Beijing, China

**Keywords:** Fabry disease, inflammatory cytokines, enzyme replacement therapy, hypertrophic cardiomyopathy, chronic kidney disease, Mainz severity score index

## Abstract

**Objectives:**

The aim of this study is to explore the expression of inflammatory cytokines (ICs) in Fabry disease (FD), the correlation between ICs and FD phenotypes, and the impact of enzyme replacement therapy (ERT) on IC expression.

**Methods:**

We recruited 67 FD patients and 44 healthy controls (HCs) and detected concentrations of the following ICs: interferon-γ, interleukin (IL)-1β, IL-2, IL-4, IL-5, IL-6, IL-8, IL-10, IL-12P70, IL-17A, IL-17F, IL-22, tumor necrosis factor (TNF)-α, and TNF-β. We also analyzed the impact of ERT on IC expression in FD patients and the relationship between IC expression and sex, genotype, phenotype, disease burden, and biomarkers.

**Results:**

Most ICs were significantly higher in FD patients than in HCs. A number of ICs were positively correlated with clinical aspects, including disease burden (Mainz Severity Score Index [MSSI]) and cardiac and renal markers. IL-8 was higher in the high MSSI (P-adj=0.026*) than in the low MSSI.

**Conclusions:**

ICs were upregulated in FD patients, indicating the role of the innate immune process in FD etiology. ERT ameliorated FD-related inflammatory activation, at least to some extent. IC expression was positively correlated with disease burden and clinical markers in FD. Our findings indicated that the inflammatory pathway may be a promising therapeutic target for FD.

## 1 Introduction

Fabry disease (FD; OMIM#301500) is a rare X-linked inherited lysosomal storage disease that is caused by mutations in the *GLA* gene (NC_000023.11), which encodes the alpha-galactosidase A protein (α-Gal A) and is located on chromosome Xq22.1 (1). *GLA* mutations lead to a deficiency of lysosomal hydrolase α-Gal A (EC 3.2.1.22) activity (2, 3) and the secondary lysosomal accumulation of globotriaosylceramide (Gb3) and other glycosphingolipids within cells. The progressive accumulation of these compounds causes cellular dysfunction, which subsequently damages multiple organs (4, 5). Plasma globotriaosylsphingosine (Lyso-Gb3) is a deacylated metabolite of Gb3 that correlates well with FD phenotype, with a higher sensitivity than Gb3 (6). Plasma Lyso-Gb3 is currently a biomarker for the diagnosis and therapeutic efficacy monitoring of FD. FD can be classified into severe (classic FD) and milder (non-classic or late-onset FD) phenotypes. Classic FD has an earlier onset; it presents as a multisystemic disorder with manifestations such as neuropathic pain, hypertrophic cardiomyopathy, renal insufficiency, and stroke. Non-classic FD is characterized by a more variable phenotype in which manifestations are predominantly limited to heart or kidney dysfunction (7–9). Enzyme replacement therapy (ERT) is the classic therapy for FD; it has been used for almost 20 years. ERT can effectively reduce Gb3 deposits in the urine, plasma, and tissues (10–12); delay multiple organ deterioration; and improve lifespan.

Inflammatory activation plays an important role in FD pathogenesis. Glycolipids, especially Gb3, can bind to toll-like receptor 4 and trigger Notch1 signaling, which in turn activates the nuclear factor kappa B signaling cascade. This process results in the production of pro-inflammatory cytokines, leading to a chronic inflammatory state and associated vasculopathy (13). The long-lasting inflammation results in fibrosis, irreversible tissue injury, and ultimately, target organ failure (14, 15). One study has also demonstrated that interleukin (IL)-1β promotes the expression of adhesion molecules, which enhances tissue infiltration and eventually results in tissue remodeling (16).

Adult classic FD patients who receive ERT treatment display reduced antioxidant and increased pro-oxidant statuses and have higher levels of the pro-inflammatory cytokines tumor necrosis factor (TNF)-α and IL-6 compared with HCs (17). These findings support the idea that oxidative stress and inflammatory activation act as major drivers of FD pathogenesis. To date, however, the potential clinical significance of inflammatory cytokine (IC) overexpression in FD remains inconclusive. In the current study, we therefore aimed to reveal the relationship between ICs and FD characteristics, such as severity and phenotype, as well as the impact of ERT on IC expression. Our findings will help to outline the inflammatory status and evaluate the inflammatory mechanisms of FD.

## 2 Methods

### 2.1 Patient enrollment and sample collection

**Figure 1:**
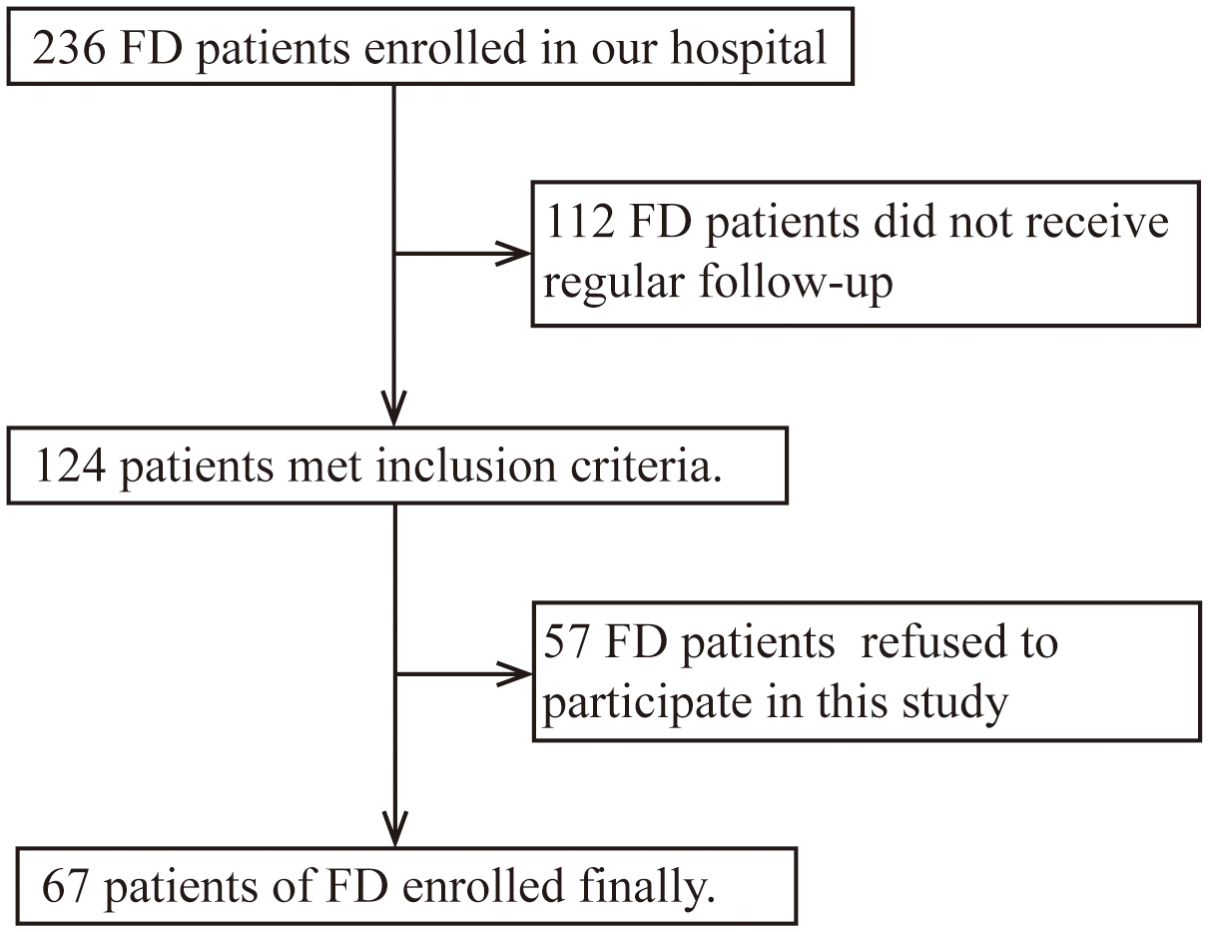
Flowchart of patient recruitment in Fabry disease.

Inclusion criteria: FD patients who underwent regular follow-up from November 2019 to June 2023 in the Neurology Department of Peking University First Hospital were enrolled. Diagnostic criteria were based on clinical history and laboratory tests (*GLA* gene, α-Gal A activity, and Lyso-Gb3) according to the Chinese consensus on FD (18). Exclusion criteria: FD patients refused to participate in this study (**Figure 1**). We selected age- and sex-matched healthy controls (HCs) from the Health Management Center of Peking University First Hospital. These patients all excluded excessive alcohol use, illegal drug use, heavy smoking, liver and kidney dysfunction, blood glucose and lipid abnormalities, infectious disease, tumors, autoimmune disease, and other disease states that might induce inflammation after medical inquiry and lab test.

Demographic and detailed medical data of all FD patients were collected. Multiple organs/systems, including the heart, kidney, peripheral nerves, and brain, were evaluated through corresponding examinations and/or scales. Peripheral whole blood was obtained from FD patients and from HCs after ruling out fever, infection, autoimmune disease, and other disease states that might induce inflammation. Samples from the FD patients were collected at baseline and/or after short-term (≤6 months) (19) and/or long-term (>6 months) ERT.

### 2.2 IC assay

Peripheral whole blood was collected in ethylenediaminetetraacetic acid tubes and immediately centrifuged at 1240 × *g* for 5 min at 4°C. Plasma was isolated and frozen at −80°C before further processing. Sample preparation and detection of cytokines—namely, interferon (IFN)-γ, IL-1β, IL-2, IL-4, IL-5, IL-6, IL-8, IL-10, IL-12P70, IL-17A, IL-17F, IL-22, TNF-α, and TNF-β—were performed using a sandwich enzyme-linked immunosorbent assay kit (914002, QuantoBio, Tianjin, China) following the manufacturer’s instructions. The cytokine concentrations of each sample were detected using a flow cytometer (BeamCyte-1026M, Jiangsu, China) and analyzed using CYTOSYS 2.0 software (Changzhou Bidako Biotechnology Co., Ltd., Changzhou, China).

### 2.3 Study design

To investigate inflammatory status in FD, we first compared plasma IC expression between naïve FD patients and HCs. We then compared each of the short-term (≤6 months) and long-term (>6 months) ERT groups with the HCs. Furthermore, we compared each of the short-term (≤6 months) and long-term (>6 months) ERT groups with naïve FD patients. Finally, we observed the trend of changes in IC expression between pre- and post-treatment states (post-short-term [ ≤ 6 months] and post-long-term [>6 months] ERT groups) only in the patients who underwent regular IC measurements during ERT.

We also analyzed the differences in IC expression between FD subgroups classified by sex, *GLA* genotype [truncated versus non-truncated mutation (20)], and clinical subtype [classic versus non-classic (7, 21)]. We then analyzed the correlations between IC expression and age of disease onset, duration, α-Gal A activity (for classic male patients only), and Lyso-Gb3.

To evaluate the clinical value of ICs, we also analyzed the correlations between IC expression and a series of disease markers, as follows: 1) disease severity [Mainz Severity Score Index [MSSI] (15, 22–24)]; 2) heart (left ventricular mass index [LVMI] (25, 26), cardiac troponin I [cTnI], and B-type natriuretic peptide [BNP]); 3) kidney (estimated glomerular filtration rate [eGFR], urine albumin-creatinine ratio [ACR], and 24-hour proteinuria [24h-P]); 4) brain [Fazekas scale to assess the brain white matter lesions [WML] on T2-weighted magnetic resonance images (27, 28)]; and 5) peripheral nerve (visual analog scale [VAS] based on the maximum pain intensity in the past 24 hours in the brief pain inventory [BPI] (29) to assess neuropathic pain; pain scores ranged from 0, which was “no pain” or “pain does not interfere,” to 10, which was “pain as bad as you can imagine” or “pain completely interferes”).

We also compared IC expression between different clinical subgroups, which were classified according to various disease conditions, as follows: low (<20) versus high (≥20) MSSI groups (23); hypertrophic cardiomyopathy (HCM; LVMI≥88 g/m² in women and ≥102 g/m² in men) versus non-HCM groups (25); chronic kidney disease (CKD; eGFR<60 mL/min/1.73 m^2^ or the presence of proteinuria) versus non-CKD groups (30, 31), mild (Fazekas<3) versus severe (Fazekas≥3) WML groups (27), neuralgia versus non-neuralgia groups, and mild (VAS<3) versus severe (VAS≥3) pain groups.

### 2.4 Statistical analysis

IBM SPSS Statistics for Windows, version 25.0 (IBM Corp, Armonk, NY, USA) was used for all analyses. The Shapiro–Wilk test was applied to determine variable distributions. Quantitative variable data with normal distributions were shown as the mean ± standard deviation, and Independent Samples t-test was used to group comparison. Quantitative variable data with non-normal distributions were shown as the median (P_25_, P_75_), and the Mann–Whitney U test was used to compare differences between subgroups. Qualitative variable data were shown as frequency, and the Chi-Square Test was applied to group comparison. Pearson’s methods were used for correlation analyses. The false discovery rate (FDR) adjusted P-value (P-adj) for multiple comparisons were used to estimate causal effects. A p-value or P-adj <0.05 was considered significant.

## 3 Results

### 3.1 Cohort characteristics

This prospective study included 67 FD patients (38 males and 29 females), derived from 47 unrelated families, as well as 44 HCs (24 males and 20 females). The mean ( ± standard deviation) age at sample collection was 39.57 ± 16.03 years for FD patients and 35.20 ± 8.74 years for HCs. Of the 67 FD patients, 3 females were asymptomatic patients; all of these asymptomatic patients had family members with symptomatic FD. The median (P_25_, P_75_) onset age and disease duration of the remaining 64 FD patients was 9.00 (7.00, 14.25) years and 23.00 (12.00, 39.50) years, respectively. The median (P_25_, P_75_) α-Gal A activity (for the 21 classic male patients only) was 0.35 (0.27, 0.44) μmol/L/h, and that of Lyso-Gb3 was 19.84 (4.56, 87.04) ng/mL. *GLA* was tested in all 47 families; 46 families had *GLA* exon mutations—comprising 24 missense mutations, 8 frameshift mutations (insertion/deletion mutations), 3 splice mutations, and 11 nonsense mutations—and 1 family had intron insertion mutations. ICs were tested in 59 patients before ERT and 16 patients after ERT (7 patients with agalsidase-α and 9 patients with agalsidase-β), in which 22 samples were collected from 3 months to 5 years after ERT. There were 26 patients in the truncated mutation group and 32 in the non-truncated mutation group, and 41 patients (31 males and 10 females) in the classic group and 18 (2 males and 16 females) in the non-classic group. The baseline demographic and clinical characteristics of the FD patients are summarized in **Table 1**.

**Table 1:** Baseline demographic and clinical characteristics.

Variables	Total sample (n = 67)	
	N/Mean/P50	%/SD/(P25,P75)
male sex	38	56.72
femle sex	29	43.28
classic phenotype	47	70.15
non-classic phenotype	20	29.85
neuropathic pain	53	79.10
hypohidrosis	32	47.76
gastrointestinal discomfort	32	47.76
tinnitus	36	53.73
hearing loss	25	37.31
angiokeratoma	22	32.84
cornea verticillate	40	59.70
retinal vascular tortuosity	21	31.34
hypertension	25	37.31
HCM	24	37.31
AF	5	7.46
CHD	4	5.97
proteinuria	31	46.27
renal insufficiency	29	43.28
dialysis or kidney transplantation	7	10.45
ischemic stroke	14	20.90
Onset age(n=64)	9.00	(7.00,14.25)
Duration(n=64)	23.00	(12.00,39.50)
α-Gal A activity(n=21)^*^	0.35	(0.27,0.44)
Lyso-Gb3(n=57)^**^	19.84	(4.56,87.04)
MSSI(n=59)	18.00	(8.00,28.00)
cTnI(n=28)	0.06	(0.01,4.60)
BNP(n=28)	96.50	(19.00,642.00)
LVMI(n=49)	91.32	(77.31,128.25)
eGFR(n=51)	96.89	(66.70,117.24)
ACR(n=44)	41.59	(8.07,162.97)
24h-P(n=30)	0.24	(0.10,1.36)
Fazekas(n=45)	2.00	(1.00,4.00)
VAS(n=59)	0.00	(0.00,3.00)

^*^α-Gal A activity (μmol/L/h) (male, classic),^**^Lyso-Gb3 (ng/mL), hypohidrosis (less or no sweat), hearing loss (including sensorineural deafness), gastrointestinal discomfort (intermittent diarrhea and constipation). 24h-P, 24-hour proteinuria; ACR, urine albumin-creatinine ratio; AF, atrial fibrillation; BNP, plasma B-type natriuretic peptide; CHD, coronary heart disease; cTnI, cardiac troponin I; HCM, hypertrophic cardiomyopathy; LVMI, left ventricular mass index; WML, cerebral white matter lesions.

### 3.2 IC expression in FD and the impact of ERT on IC expression

**Figure 2:**
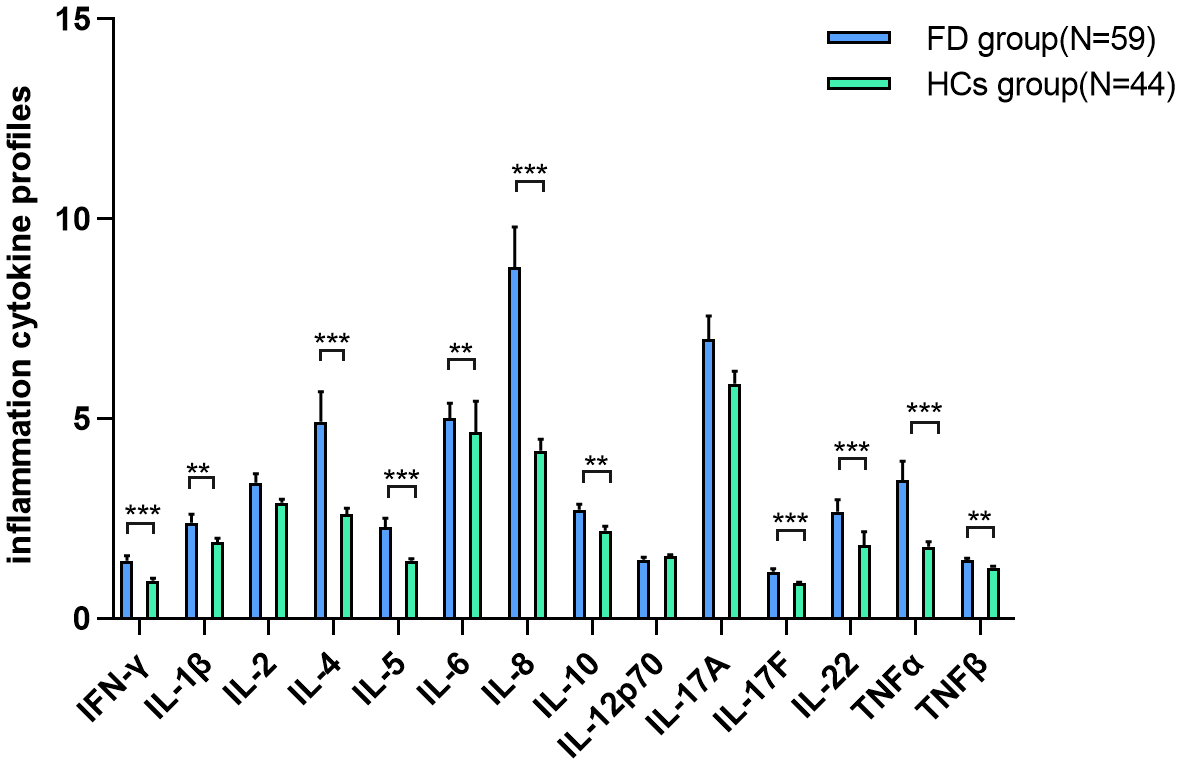
IC expression in FD IFN-γ, IL-1β, IL-4, IL-5, IL-6, IL-8, IL-10, IL-17F, IL-22, TNF-α, and TNF-β expression was significantly higher in FD patients before the initiation of ERT than in the HC group. Data represent the mean±standard error of the mean. ** P-adj<0.01; *** P-adj<0.001. FD, Fabry disease; IC, inflammatory cytokine; IFN-γ, interferon-gamma; IL, interleukin; TNF, tumor necrosis factor.

As shown in **Table 2** and **Figure 2**, IFN-γ (Z=−4.667, P-adj<0.001), IL-1β (Z=−3.214, P-adj=0.002), IL-4 (Z=−3.800, P-adj<0.001), IL-5 (Z=−3.987, P-adj<0.001), IL-6 (Z=−2.887, P-adj=0.005), IL-8 (Z=−5.273, P-adj<0.001), IL-10 (Z=−3.080, P-adj=0.003), IL-17F (Z=−3.727, P-adj<0.000), IL-22 (Z=−4.060, P-adj<0.001), TNF-α (Z=−4.274, P-adj<0.001), and TNF-β (Z=−2.877, P-adj=0.005) expression was significantly higher in 59 FD patients before the initiation of ERT than in the HC group (N=44). There were no differences in IL-2, IL-12P70 and IL-17A expression between the FD and HC groups.

**Table 2:** IC expression in FD.

	FD	HCs	Z	P	P-adj
sex	33/26	24/20			
age	39.54±16.56	35.20±8.74			
IFN-γ	1.18(0.99,1.68)	0.83(0.75,1.01)	-4.667	0.000	<0.001***
IL-1β	2.09(1.76,2.80)	1.72(1.50,1.93)	-3.214	0.001	0.002**
IL-2	3.19(2.55,3.97)	2.86(2.53,3.22)	-1.993	0.046	0.054
IL-4	3.04(2.40,4.88)	2.34(2.04,2.60)	-3.800	0.000	<0.001***
IL-5	1.72(1.35,2.26)	1.39(1.14,1.63)	-3.987	0.000	<0.001***
IL-6	4.45(3.08,5.44)	3.25(2.84,3.89)	-2.887	0.004	0.005**
IL-8	5.97(4.76,10.60)	3.45(3.06,4.74)	-5.273	0.000	<0.001***
IL-10	2.54(1.95,3.26)	2.01(1.64,2.43)	-3.080	0.002	0.003**
IL-12p70	1.53(1.22,1.83)	1.60(1.49,1.69)	-0.483	0.629	0.629
IL-17A	6.59(4.16,8.61)	5.54(4.74,6.89)	-1.340	0.180	0.194
IL-17F	0.95(0.88,1.29)	0.85(0.76,0.90)	-3.727	0.000	<0.001***
IL-22	1.86(1.28,3.36)	1.28(1.10,1.55)	-4.060	0.000	<0.001***
TNFα	2.10(1.75,4.27)	1.56(1.39,1.90)	-4.274	0.000	<0.001***
TNFβ	1.41(1.22,1.66)	1.26(1.14,1.36)	-2.877	0.004	0.005**

*P-adj<0.05; **P-adj<0.01; ***P-adj<0.001. FD, Fabry disease; HCs, healthy controls; IC, inflammatory cytokine; IFN-γ, interferon-gamma; IL, interleukin; TNF, tumor necrosis factor.

IFN-γ (Z=−3.018, P-adj=0.008), IL-1β (Z=−2.714, P-adj=0.013), IL-4 (Z=−3.069, P-adj=0.008), IL-5 (Z=−2.993, P-adj=0.008), IL-6 (Z=−2.308, P-adj=0.029), IL-8 (Z=−2.841, P-adj=0.011), IL-10 (Z=−2.511, P-adj=0.019), IL-17F (Z=−2.967, P-adj=0.008), IL-22 (Z=−2.663, P-adj=0.014), and TNF-α (Z=−3.044, P-adj=0.008) expression was significantly higher in the short-term ERT group (N=8) than in the HC group (N=44). IFN-γ (Z=−3.598, P-adj=0.001), IL-5 (Z=−2.326, P-adj=0.035), IL-8 (Z=−3.089, P-adj=0.006), IL-17F (Z=−3.834, P-adj=0.001), IL-22 (Z=−3.380, P-adj=0.003), and TNF-α (Z=−3.016, P-adj=0.006) expression was significantly higher in the long-term ERT group (N=14) than in the HC group (N=44), whereas IL-2 (Z=−2.399, P-adj=0.033) and IL-17A (Z=−4.228, P-adj<0.001) expression was significantly lower in the long-term ERT group than in the HC group (**Table 3**).

**Table 3:** Impact of ERT on IC expression.

	short-term ERT group	HCs	Z	P	P-adj	long-term ERT group	HCs	Z	P	P-adj	short-term ERT group	FD	Z	P	P-adj	long-term ERT group	FD	Z	P	P-adj
sex	5/3	24/20				9/5	24/20				5/3	33/26				9/5	33/26			
age	48.50±15.70	35.20±8.74				40.50±13.21	35.20±8.74				48.50±15.70	39.54±16.56				40.50±13.21	39.54±16.56			
IFN-γ	1.87(1.29,2.00)	0.83(0.75,1.01)	-3.018	0.003	0.008**	1.42(1.00,1.61)	0.83(0.75,1.01)	-3.598	0.000	0.001**	1.87(1.29,2.00)	1.18(0.99,1.68)	-1.838	0.066	0.183	1.42(1.00,1.61)	1.18(0.99,1.68)	-0.442	0.659	0.927
IL-1β	3.04(2.41,3.81)	1.72(1.50,1.93)	-2.714	0.007	0.013*	1.84(1.63,2.30)	1.72(1.50,1.93)	-1.090	0.276	0.297	3.04(2.41,3.81)	2.09(1.76,2.80)	-1.982	0.047	0.166	1.84(1.63,2.30)	2.09(1.76,2.80)	-1.282	0.200	0.559
IL-2	3.84(2.68,5.26)	2.86(2.53,3.22)	-1.851	0.064	0.079	2.20(1.26,2.82)	2.86(2.53,3.22)	-2.399	0.016	0.033*	3.84(2.68,5.26)	3.19(2.55,3.97)	-0.957	0.338	0.486	2.20(1.26,2.82)	3.19(2.55,3.97)	-2.943	0.003	0.023*
IL-4	9.07(3.41,11.01)	2.34(2.04,2.60)	-3.069	0.002	0.008**	3.02(2.22,4.51)	2.34(2.04,2.60)	-1.872	0.061	0.095	9.07(3.41,11.01)	3.04(2.40,4.88)	-2.146	0.032	0.166	3.02(2.22,4.51)	3.04(2.40,4.88)	-0.42	0.674	0.927
IL-5	3.51(1.88,4.52)	1.39(1.14,1.63)	-2.993	0.003	0.008**	1.75(1.26,2.47)	1.39(1.14,1.63)	-2.326	0.020	0.035*	3.51(1.88,4.52)	1.72(1.35,2.26)	-2.069	0.039	0.166	1.75(1.26,2.47)	1.72(1.35,2.26)	-0.378	0.705	0.927
IL-6	4.72(4.22,8.07)	3.25(2.84,3.89)	-2.308	0.021	0.029*	3.92(3.23,4.85)	3.25(2.84,3.89)	-1.690	0.091	0.116	4.72(4.22,8.07)	4.45(3.08,5.44)	-0.822	0.411	0.486	3.92(3.23,4.85)	4.45(3.08,5.44)	-0.61	0.542	0.927
IL-8	8.10(5.78,9.84)	3.45(3.06,4.74)	-2.841	0.005	0.011*	6.22(4.09,9.07)	3.45(3.06,4.74)	-3.089	0.002	0.006**	8.10(5.78,9.84)	5.97(4.76,10.60)	-0.812	0.417	0.486	6.22(4.09,9.07)	5.97(4.76,10.60)	-0.21	0.834	0.927
IL-10	3.58(2.38,4.48)	2.01(1.64,2.43)	-2.511	0.012	0.019*	2.16(1.49,2.59)	2.01(1.64,2.43)	-0.018	0.986	0.986	3.58(2.38,4.48)	2.54(1.95,3.26)	-1.76	0.078	0.183	2.16(1.49,2.59)	2.54(1.95,3.26)	-1.997	0.046	0.214
IL-12p70	1.70(0.85,2.05)	1.60(1.49,1.69)	-0.583	0.560	0.603	1.35(1.19,1.69)	1.60(1.49,1.69)	-1.563	0.118	0.138	1.70(0.85,2.05)	1.53(1.22,1.83)	-0.309	0.757	0.787	1.35(1.19,1.69)	1.53(1.22,1.83)	-0.645	0.519	0.927
IL-17A	4.90(3.30,10.19)	5.54(4.74,6.89)	-0.279	0.780	0.780	2.94(2.22,3.44)	5.54(4.74,6.89)	-4.288	0.000	<0.001***	4.90(3.30,10.19)	6.59(4.16,8.61)	-0.271	0.787	0.787	2.94(2.22,3.44)	6.59(4.16,8.61)	-3.489	0.000	0.007**
IL-17F	1.34(1.10,1.81)	0.85(0.76,0.90)	-2.967	0.003	0.008**	1.11(0.95,1.28)	0.85(0.76,0.90)	-3.834	0.000	0.001**	1.34(1.10,1.81)	0.95(0.88,1.29)	-2.011	0.044	0.166	1.11(0.95,1.28)	0.95(0.88,1.29)	-1.31	0.190	0.559
IL-22	3.07(1.93,4.43)	1.28(1.10,1.55)	-2.663	0.008	0.014*	1.87(1.67,2.54)	1.28(1.10,1.55)	-3.380	0.001	0.003**	3.07(1.93,4.43)	1.86(1.28,3.36)	-1.257	0.209	0.365	1.87(1.67,2.54)	1.86(1.28,3.36)	-0.287	0.774	0.927
TNFα	4.78(2.51,5.42)	1.56(1.39,1.90)	-3.044	0.002	0.008**	2.43(1.68,3.07)	1.56(1.39,1.90)	-3.016	0.003	0.006**	4.78(2.51,5.42)	2.10(1.75,4.27)	-1.653	0.098	0.197	2.43(1.68,3.07)	2.10(1.75,4.27)	-0.175	0.861	0.927
TNFβ	1.61(1.19,2.58)	1.26(1.14,1.36)	-1.826	0.068	0.079	1.36(1.15,1.88)	1.26(1.14,1.36)	-1.726	0.084	0.116	1.61(1.19,2.58)	1.41(1.22,1.66)	-0.851	0.395	0.486	1.36(1.15,1.88)	1.41(1.22,1.66)	-0.028	0.978	0.978

*P-adj<0.05; **P-adj<0.01; ***P-adj<0.001. ERT, enzyme replacement therapy; IC, inflammatory cytokine; IFN, interferon; IL, interleukin; TNF, tumor necrosis factor.

There were no differences in 14 ICs expression between the short-term ERT group and the naïve patients group. IL-2 (Z=−2.943, P-adj=0.023) and IL-17A (Z=−3.489, p=0.007) expression were significantly lower in the long-term ERT group(N=14) than in the naïve patients group(N=59) (**Table 3**, showing data with significant differences only).

### 3.3 Correlations between ICs and disease markers

We analyzed the correlations between IC expression and disease characteristics and biomarkers among 59 patients before ERT initiation. IC expression levels were not significantly associated with onset age, duration, α-Gal A activity, or plasma Lyso-Gb3.

**Figure 3:**
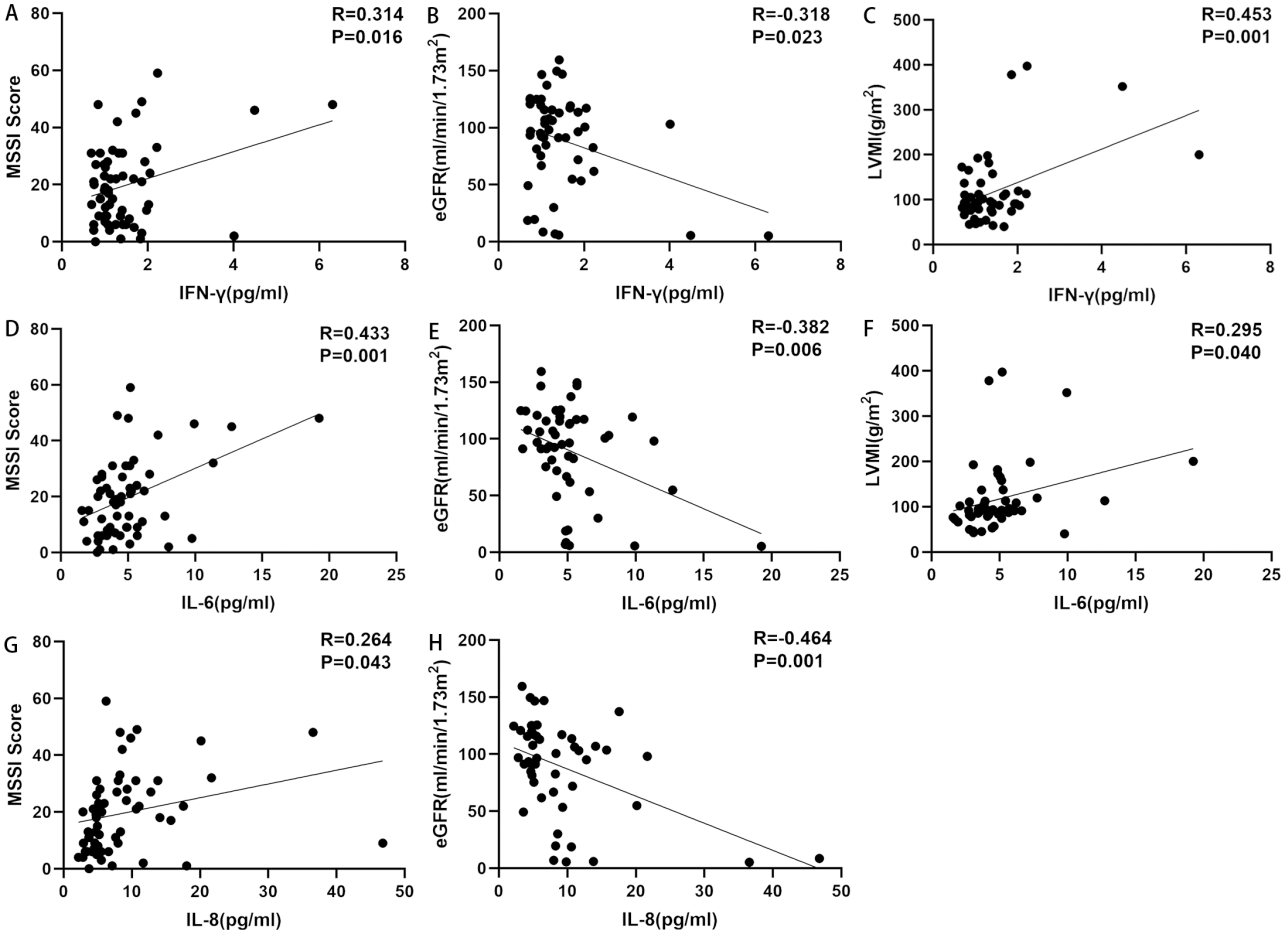
Correlations between ICs and disease markers. **(A)** Correlation between IFN-γ and MSSI. IFN-γ was positively correlated with MSSI. **(B)** Correlation between IFN-γ and eGFR. IFN-γ was negatively correlated with eGFR. **(C)** Correlation between IFN-γ and LVMI. IFN-γ was positively correlated with LVMI. **(D)** Correlation between IL-6 and MSSI. IL-6 was positively correlated with MSSI. **(E)** Correlation between IL-6 and eGFR. IL-6 was negatively correlated with eGFR. **(F)** Correlation between IL-6 and LVMI. IL-6 was positively correlated with LVMI. **(G)** Correlation between IL-8 and MSSI. IL-8 was positively correlated with MSSI. **(H)** Correlation between IL-8 and eGFR. IL-8 was negatively correlated with eGFR. eGFR, estimated glomerular filtration rate; ICs, inflammatory cytokines; IFN-γ, interferon-gamma; IL, interleukin; LVMI, left ventricular mass index; MSSI, Mainz Severity Score Index.

The results of the correlation analyses are shown in **Table 4**. IFN-γ (r=0.314, p=0.016), IL-1β (r=0.267, p=0.041), IL-2 (r=0.289, p=0.026), IL-4 (r=0.278, p=0.033), IL-6 (r=0.433, p=0.001), IL-8 (r=0.264, p=0.043), IL-17F (r=0.357, p=0.006), and IL-22 (r=0.294, p=0.024) were positively correlated with MSSI. IFN-γ (r=0.453, p=0.001), IL-6 (r=0.295, p=0.04), and IL-17F (r=0.413, p=0.003) were positively correlated with LVMI. IFN-γ (r=0.499, p=0.007), IL-8 (r=0.424, p=0.025), IL-17F (r=0.661, p=0.000), and TNF-α (r=0.424, p=0.025) were positively correlated with BNP, whereas IL-1β (r=−0.408, p=0.031) was negatively correlated with BNP. Both IFN-γ (r=0.573, p=0.001) and IL-17F (r=0.653, p=0.000) were positively correlated with cTNI, whereas IL-1β (r=−0.433, p=0.021) was negatively correlated with cTNI. IFN-γ (r=−0.318, p=0.023), IL-2 (r=−0.309, p=0.028), IL-6 (r=−0.382, p=0.006), IL-8 (r=−0.464, p=0.001), and TNF-α (r=−0.293, p=0.037) were negatively correlated with eGFR. IFN-γ (r=0.393, p=0.008), IL-1β (r=0.494, p=0.001), IL-2 (r=0.496, p=0.001), IL-4 (r=0.423, p=0.004), IL-5 (r=0.380, p=0.011), IL-6 (r=0.509, p=0.000), IL-8 (r=0.496, p=0.001), IL-10 (r=0.374, p=0.012), IL-12p70 (r=0.352, p=0.019), IL-17A (r=0.489, p=0.001), IL-17F (r=0.439, p=0.003), IL-22 (r=0.397, p=0.008), and TNF-α (r=0.367, p=0.014) were all positively correlated with ACR. IFN-γ (r=0.768, p=0.000), IL-1β (r=0.914, p=0.000), IL-2 (r=0.899, p=0.000), IL-4 (r=0.772, p=0.000), IL-5 (r=0.803, p=0.000), IL-6 (r=0.737, p=0.000), IL-8 (r=0.539, p=0.002), IL-10 (r=0.658, p=0.000), IL-17A (r=0.871, p=0.000), IL-17F (r=0.721, p=0.000), IL-22 (r=0.776, p=0.000), and TNF-α (r=0.908, p=0.000) were all positively correlated with 24h-P. IL-6 (r=−0.262, p=0.045) was negatively correlated with BPI. By contrast, none of the cytokines were significantly associated with Fazekas scores (**Figure 3**, data supplement in **Table 4**).

**Table 4:** Correlations of IC expression with disease markers.

Variables		MSSI(n=59)	LVMI(n=49)	BNP(n=28)	cTNI(n=28)	eGFR(n=51)	ACR(n=44)	24h-P(n=30)	Fazekas(n=45)	BPI(n=59)	onset age(n=56)	duration(n=56)	α-GaL A activity(n=21)	Lyso-Gb3(n=57)
IFN-γ	r	0.314*	0.453**	0.499**	0.573**	-0.318*	0.393**	0.768**	0.048	-0.165	-0.041	0.075	-0.064	0.000
	P	0.016	0.001	0.007	0.001	0.023	0.008	0.000	0.754	0.212	0.767	0.582	0.784	1.000
IL-1β	r	0.267*	0.185	-0.408*	-0.433*	-0.220	0.494**	0.914**	0.075	-0.058	0.020	0.045	-0.010	-0.035
	P	0.041	0.204	0.031	0.021	0.121	0.001	0.000	0.624	0.662	0.886	0.740	0.965	0.798
IL-2	r	0.289*	0.187	0.354	0.221	-0.309*	0.496**	0.899**	0.152	-0.163	0.073	0.137	0.040	-0.069
	P	0.026	0.199	0.064	0.257	0.028	0.001	0.000	0.319	0.217	0.594	0.316	0.863	0.608
IL-4	r	0.278*	0.264	0.165	0.077	-0.164	0.423**	0.772**	0.025	-0.090	-0.093	0.116	-0.049	0.120
	P	0.033	0.067	0.400	0.696	0.249	0.004	0.000	0.869	0.497	0.494	0.393	0.832	0.374
IL-5	r	0.213	0.160	-0.042	-0.093	-0.135	0.38*	0.803**	-0.002	-0.057	-0.070	0.092	0.039	0.090
	P	0.105	0.274	0.834	0.638	0.344	0.011	0.000	0.989	0.669	0.607	0.502	0.866	0.507
IL-6	r	0.433**	0.295*	0.260	0.364	-0.382**	0.509**	0.737**	0.160	-0.262*	-0.074	0.226	-0.210	0.137
	P	0.001	0.040	0.181	0.057	0.006	0.000	0.000	0.294	0.045	0.588	0.094	0.360	0.311
IL-8	r	0.264*	0.157	0.424*	0.208	-0.464**	0.496**	0.539**	-0.045	-0.188	0.078	0.031	0.022	0.061
	P	0.043	0.281	0.025	0.288	0.001	0.001	0.002	0.770	0.153	0.568	0.818	0.925	0.653
IL-10	r	0.183	0.249	0.362	0.157	-0.148	0.374*	0.658**	-0.006	0.013	0.123	0.061	0.172	0.008
	P	0.165	0.085	0.058	0.425	0.300	0.012	0.000	0.969	0.923	0.365	0.653	0.456	0.950
IL-12p70	r	0.068	-0.013	-0.016	-0.131	-0.083	0.352*	0.283	0.176	-0.089	0.117	-0.024	-0.385	-0.052
	P	0.609	0.928	0.934	0.507	0.562	0.019	0.130	0.248	0.500	0.392	0.861	0.085	0.700
IL-17A	r	0.234	0.111	-0.187	-0.156	-0.198	0.489**	0.871**	0.143	-0.156	0.017	0.178	0.068	-0.064
	P	0.074	0.449	0.340	0.427	0.165	0.001	0.000	0.348	0.238	0.902	0.188	0.770	0.638
IL-17F	r	0.357**	0.413**	0.661**	0.653**	-0.267	0.439**	0.721**	0.131	-0.141	-0.059	0.165	-0.063	0.163
	P	0.006	0.003	0.000	0.000	0.058	0.003	0.000	0.390	0.286	0.664	0.224	0.787	0.226
IL-22	r	0.294*	0.274	0.302	0.292	-0.156	0.397**	0.776**	0.029	-0.116	-0.069	0.071	-0.058	0.095
	P	0.024	0.057	0.118	0.131	0.274	0.008	0.000	0.852	0.383	0.614	0.601	0.803	0.481
TNFα	r	0.190	0.190	0.424*	0.273	-0.293*	0.367*	0.908**	0.053	-0.105	0.022	-0.008	0.017	-0.041
	P	0.150	0.192	0.025	0.159	0.037	0.014	0.000	0.730	0.429	0.870	0.956	0.942	0.762
TNFβ	r	0.058	-0.063	0.181	-0.014	-0.254	0.243	0.176	0.014	-0.210	0.139	0.071	-0.035	-0.048
	P	0.665	0.665	0.356	0.944	0.072	0.112	0.351	0.925	0.111	0.306	0.601	0.881	0.724

**p<0.01 (two-tailed); *p<0.05 (two-tailed). 24h-P, 24-hour proteinuria; ACR, urine albumin-creatinine ratio; BNP, B-type natriuretic peptide; BPI, brief pain inventory; cTnI, cardiac troponin I; eGFR, estimated glomerular filtration rate; IC, inflammatory cytokine; IFN, interferon; IL, interleukin; LVMI, left ventricular mass index; LVMI=LVM/BSA, where LV mass=0.8×1:04×[(IVS+LVID+PWT)^3^−LVID^3^]+0.6g and BSA(m^2^)=(height(cm)×weight(kg)/3600)^1/2^; MSSI, Mainz Severity Score Index; TNF, tumor necrosis factor.

### 3.4 IC expression between different FD subgroups

There were no significant differences in IC expression between men and women, between non-truncated mutation group and truncated mutation group, or between patients with classic and non-classic FD.

IL-8 (Z=−3.112, P-adj=0.026) expression was higher in the high MSSI group (N=28) than in the low MSSI group (N=31). There were no significant differences in IC expression between the HCM and non-HCM groups, the CKD and non-CKD groups, mild and severe WML groups, neuralgia and non-neuralgia groups, or mild and severe pain groups (**Table 5**, showing data with significant differences only).

**Table 5:** IC expression between different FD subgroups.

	low MSSI score group	high MSSI score group	Z	P	P-adj
sex	11/20	22/6			
age	33.97±17.61	45.71±13.00			
IFN-γ	1.11(1.00,1.49)	1.28(0.91,1.86)	-0.661	0.509	0.826
IL-1β	2.08(1.73,2.71)	2.22(1.79,2.83)	-0.410	0.682	0.868
IL-2	2.92(2.25,3.85)	3.50(2.75,3.98)	-1.488	0.137	0.479
IL-4	3.04(2.38,3.99)	3.05(2.42,5.78)	-0.964	0.335	0.685
IL-5	1.68(1.48,2.19)	1.84(1.30,2.68)	-0.015	0.988	0.988
IL-6	4.05(2.91,5.07)	5.09(3.72,6.07)	-2.209	0.027	0.190
IL-8	4.93(3.73,7.62)	8.45(5.41,11.00)	-3.112	0.002	0.026*
IL-10	2.52(1.91,3.11)	2.55(2.06,3.60)	-0.554	0.580	0.826
IL-12p70	1.49(1.16,1.85)	1.55(1.25,1.83)	-0.084	0.933	0.988
IL-17A	5.90(3.19,7.97)	7.10(5.00,9.95)	-1.799	0.072	0.336
IL-17F	0.95(0.86,1.18)	1.01(0.89,1.48)	-0.949	0.343	0.685
IL-22	1.78(1.28,3.16)	2.03(1.29,4.25)	-0.979	0.327	0.685
TNFα	2.18(1.75,4.05)	2.09(1.75,4.29)	-0.030	0.976	0.988
TNFβ	1.36(1.10,1.65)	1.49(1.26,1.72)	-0.539	0.590	0.826

*P-adj<0.05. FD, Fabry disease; IC, inflammatory cytokine; MSSI, Mainz Severity Score Index; IFN, interferon; IL, interleukin; TNF, tumor necrosis factor.

## 4 Discussion

Evidence of inflammatory activation in FD has been reported in previous research. Uçeyler et al (32). reported that peripheral blood mononuclear cells (PBMC) from men with FD had higher baseline IL-1β gene expression compared to healthy men. De Francesco et al. (16) revealed that IL-1β and TNF-α were increased in unstimulated PBMC from 29 FD patients with acroparesthesia compared with HCs. And the supernatants of 24 h PBMC cultures had a significant increase in secreted IL-1β and IL-6. Dendritic cells and Monocytic population from Fabry patients’ PBMC showed a higher basal level of IL-1β compared to controls. Furthermore, a tendency toward a reduced percentage of invariant natural killer T cells producing IL-4 has been reported in naïve FD patients (33). Our results further indicate that diffuse inflammatory pathway activation occurs in FD, involving pro-inflammatory cytokines such as IL-1β, IL-5, IL-6, IL-8, IL-12, IL-17A, IL-17F, IL-22, TNF-α, TNF-β, and IFN-γ (produced by T cells and monocytes/macrophages) and anti-inflammatory cytokines such as IL-4 and IL-10 (produced by monocytes/macrophages) (34, 35). Of these cytokines, some already been previously reported, such as IL-1β and TNF-α. De Francesco et al (16). finding suggests that excess amount Gb3 could in fact interact with TLR4, consequently triggering a signaling cascade that leads to produce IL-1β and TNF-α.

ICs have a crucial role in the pathophysiology of FD-associated pain. Lyso-Gb3 deposition can reportedly impair calcium-activated potassium channel subunit alpha-1 activity and activate the Notch1 signaling pathway, thus inducing cutaneous nociceptor sensitization and pain attacks (36). In a cohort of 67 FD patients reported by Nurcan et al. (32), male FD patients with pain had higher TNF and IL-10 mRNA expression than those without pain, but had lower IL-4 mRNA expression. Moreover, classic male FD patients with pain had higher TNF mRNA expression than those without pain. In the present study, the presence of neuralgia or painful attacks in FD did not have a clear effect on IC expression. Even when patients were further divided by maximum pain intensity in the previous 24 hours, there were no changes in IC expression levels. This disparity in findings might be the result of differences in patient stratification methods and/or sample origins (*in vitro* peripheral blood mononuclear cells versus plasma).

ICs may be a potential candidate biomarker reflecting disease burden and severity in FD. In one study of 36 classic FD patients, IL-6 and TNF-α levels were positively correlated with MSSI (37). In the present cohort, IFN-γ, IL-1β, IL-2, IL-4, IL-6, IL-8, IL-17F, and IL-22 all correlated with MSSI. Moreover, IL-8 was higher in the high MSSI group than in the low MSSI group. FD is characterized by proteinuria and progressive renal insufficiency (14). A previous study reported that 18 FD patients with renal dysfunction had higher levels of TNF, TNF receptor 1, and TNF receptor 2 (15). Cardiomyopathy with concentric hypertrophy and diastolic dysfunction is currently the most common cause of death in patients with FD (15), and histological myocarditis is detected in up to 56% of patients with cardiomyopathy (38). Chien et al. (39) reported that serum IL-18 is slightly increased in patients with left ventricular hypertrophy alone, and is even higher in FD patients with left ventricular hypertrophy carrying the IVS4 + 919 G>A mutation. Although it has already been described how inflammatory cytokines contribute to renal damage, and the cardiac fibrosis, there is currently rare conclusion on the statistical correlation analysis between ICs and these organ specific markers. The majority of the related studies in FD were binary classifications. Our study further reveals the correlation between IC and organ specific biomarkers for monitoring the disease. The expression of many ICs positively correlated with ACR, 24h-P, and eGFR, thus indicating the diffuse interaction of ICs with renal phenotypes. IFN-γ, IL-6, and IL-17F were positively correlated with LVMI, indicating that IC elevation may play a key role in the development of myocardial inflammatory damage and remodeling in FD.

**Figure 4:**
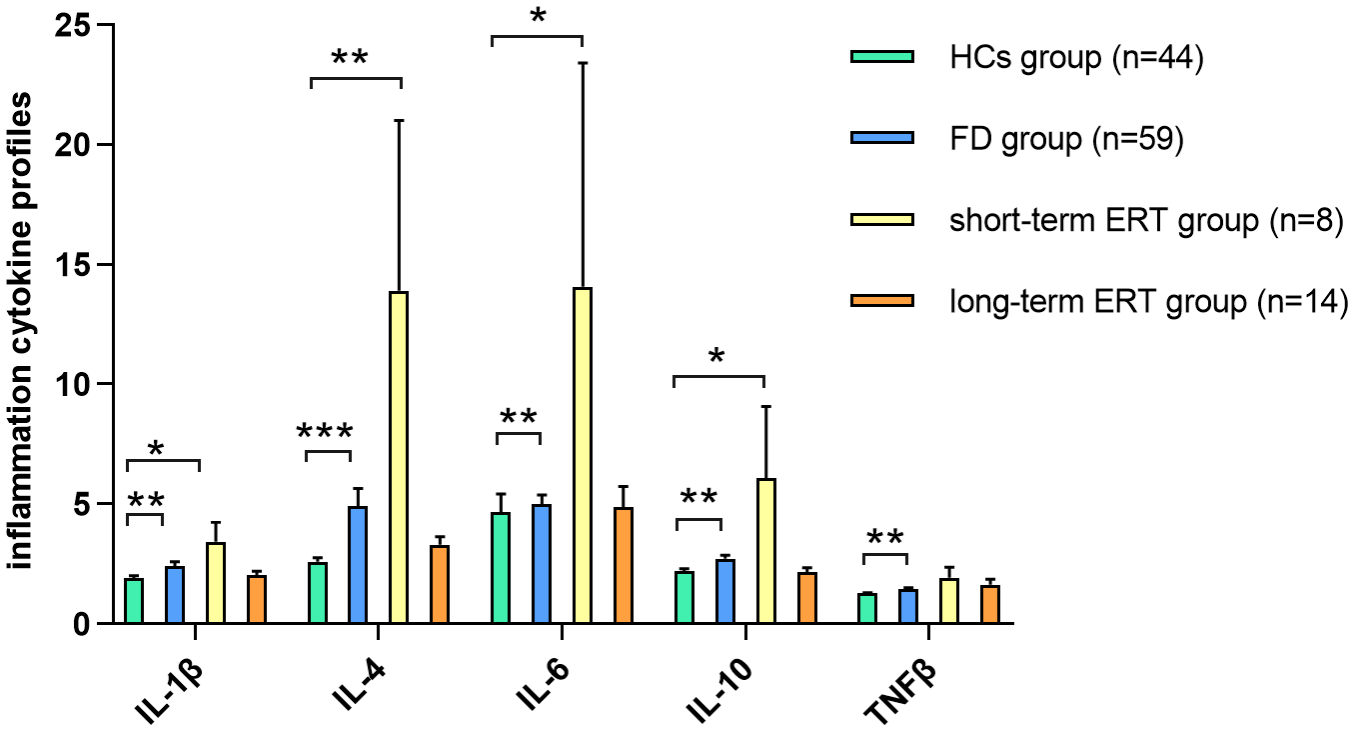
IC expression between short-/long-term ERT and FD naïve groups TNF-β expression in FD was restored in FD patients after short-term (≤6 months) ERT compared with HCs. IL-1β, IL-4, IL-6, and IL-10 expression was restored in FD patients after long-term (>6 months) ERT compared with HCs. Data represent the mean±standard error of the mean. *P-adj<0.05; **P-adj<0.01; ***P-adj<0.001. FD, Fabry disease; ERT, enzyme replacement therapy; HCs, healthy controls; IC, inflammatory cytokine; IL, interleukin; TNF, tumor necrosis factor.

**Figure 5:**
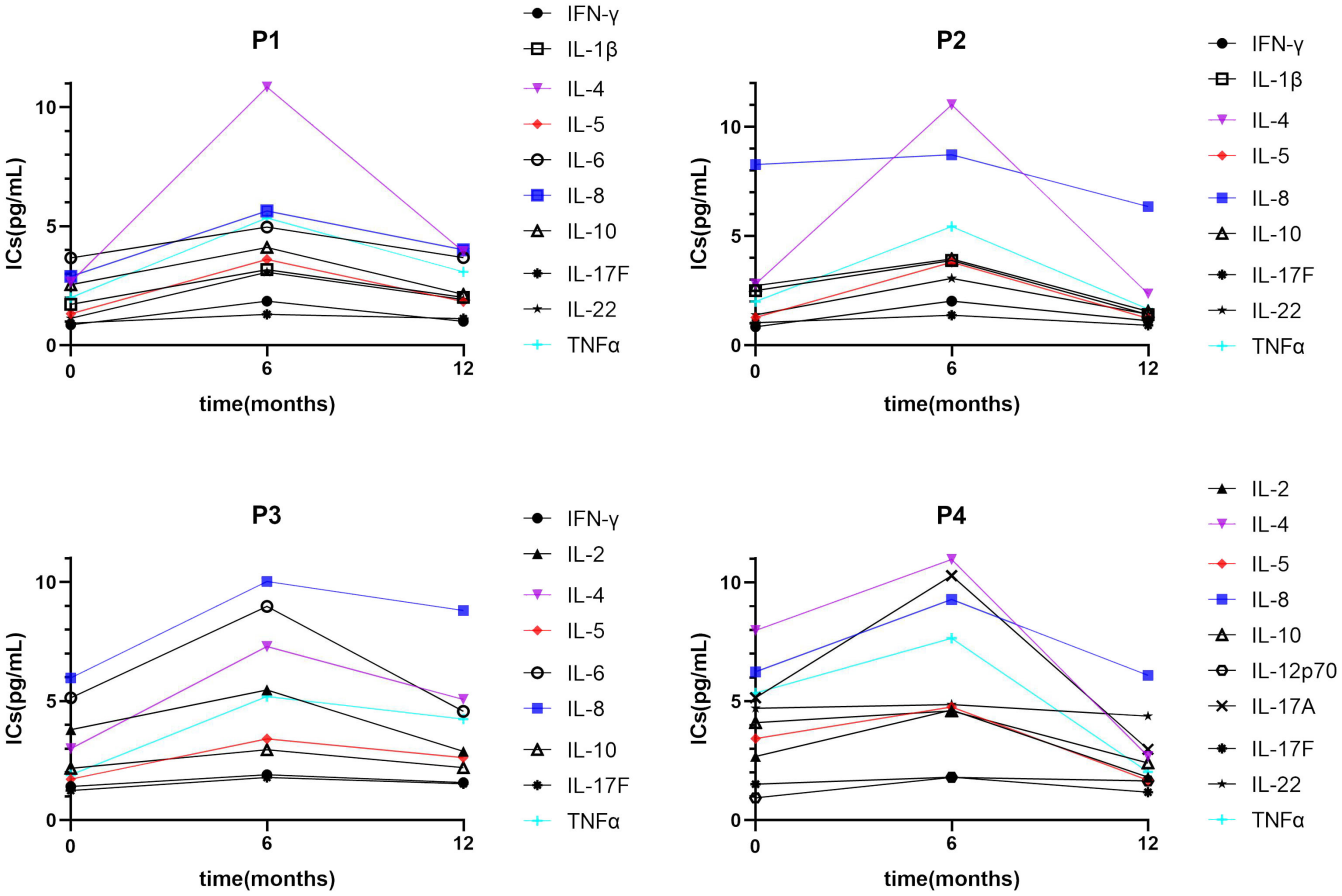
A trend of IC expression between pre-treatment and short-/long-term ERT. Many ICs show a trend of transient upward on 6 months ERT and then downward on 12 months ERT. IC, inflammatory cytokine; IL, interleukin.

ERT can ameliorate the inflammatory status of FD; In a cohort of 30 FD patients who carried the IVS4 + 919 G>A mutation and developed left ventricular hypertrophy (39), IL-18 levels were decreased after ERT, and IL-18 was significantly increased in the cardiac biopsies of patients with poor ERT responses. It is generally accepted that Lyso-Gb3 is a good biomarker which is regular monitored during ERT. In van Breemen’s research (19), 43 Fabry patients presented with classical Fabry disease and received a minimum of 12 months of ERT. They observed ERT led to prominent reductions of plasma Lyso-Gb3 in Fabry males within 3 months, and stabilized after 6 months. Therefore, In the current study, we selected 6 months as the borderline to observe the trend of changes in ICs after ERT. TNF-β expression was restored after short-term (≤6 months) ERT, and the expression of IL-1β, IL-4, IL-6, and IL-10 was restored after long-term (>6 months) ERT (**Figure 4**). Furthermore, IL-2 and IL-17A showed a downward trend with long-term treatment compared with HCs. Similarly, IL-2 and IL-17A restored to pre-treatment levels after long-term treatment compared with naïve patients. In the four individuals who underwent regular IC detection pre- and post-ERT, we observed many ICs show a trend of transient upward on 6 months ERT and then downward on 12 months ERT (**Figure 5**), similar as shown in **Figure 4**. Plasma lyso-Gb3 can activate podocyte Notch1 signaling pathway and upregulate IC expression (13). ERT, to a large extent, induced reversal of Fabry nephropathy-associated coordinated transcriptional alterations to levels comparable with normal controls over time. No significance was yielded when comparing the molecular states between patients treated with 10 and 5 years of ERT indicating that long term ERT helps to maintain good condition including innate immune system (40). Nevertheless, Neto et al. (37) reported higher serum IL-6 and TNF-α levels in patients treated with ERT than those not treated, which was similar as our result. We therefore speculate that the massive Gb3 released from cells into circulation on the initiation of ERT would lead to the transient upregulation of immune status. With the continued ERT and residual Gb3, the inflammatory response tends to stabilize.

IC expression does not appear to be directly related to disease biomarkers; their relationships may thus be complex. In our cohort, there were no correlations between the expression of any ICs and α-Gal A. In a previous study of 76 patients, there were no correlations between α-Gal A and IL-10, IL-1β, IL-1α, or TNF-α (41), consistent with our results. We further explored the potential correlations between IC expression and Lyso-Gb3 in the present study, which revealed no significant findings. However, Biancini et al. (42) demonstrated that IL-6 and TNF-α were increased in patients. IL-6 was positively correlated with Gb3. Furthermore, Üçeyler et al. (32) reported that Lyso-Gb3 is markedly enhanced in peripheral blood mononuclear cells from FD patients after TNF-α treatment, thus suggesting that overactive inflammation may also accelerate Lyso-Gb3 deposits within cells.

## 5 Limitations

In the present study, there were few paired pre- and post-ERT samples. A larger sample size would enable the further exploration of the effects of ERT on IC expression—and of their correlation with clinical biomarkers and disease burden scores—in FD. Additionally, our study involved the expression of multiple ICs, which may interact in a complex network. The upstream mRNA regulation of these ICs might provide more detailed clues about inflammatory pathway activation in FD.

## 6 Conclusions

In FD patients, the expression of ICs was widely upregulated. ERT helped to ameliorate inflammatory activation in FD. With the prolongation of ERT, some ICs restored, indicating the necessity of long-term ERT. Inflammatory status was positively correlated with disease burden and clinical markers in FD. By contrast, there were no significant correlations between inflammatory status and disease markers. Together, our findings indicate that the inflammatory pathway may be a promising therapeutic target for FD.

## Data Availability

The original contributions presented in the study are included in the article/supplementary material. Further inquiries can be directed to the corresponding author/s.
